# The Finding of Greely

**Published:** 1885-05

**Authors:** 


### The Finding of Greely.

From an account by Ensign Harlow, of the Greely Relief ship *Thetis* in the May *Century*, we quote this description of Greely's condition when found: "The launch whistled frequently as she steamed along, and we knew afterwards that the sound was heard by those who lay in the tent, which was partly blown down. Brainard and Long succeeded in creeping out from under its folds, and crawled to the top of a hill near by, from which was visible the coast towards Cape Sabine. At first nothing was seen by them; and Brainard returned to the tent, telling by the silent despair of his face that 'there was no hope.' The survivors discussed the probable cause of the noise, and decided that it was the wind blowing over the edge of a tin can. Meanwhile Long crept higher up the hill and watched attentively in the direction from which the sound had apparently come. A small, black object met his gaze. It might be a rock, but none had been seen there before. A thin, white cloud appeared above it; his ear caught the welcome sound, and the poor fellow knew that relief had come. In the ecstasy of his joy he raised the signal flag, which had blown down. It was a sad, pitiable object,—the back of a white flannel undershirt, the leg of a pair of drawers, and a piece of blue bunting tacked to an oar. The effort proved to much for him, and he sank exhausted on the rocks. It was enough for the relief party; they saw him, whistled again, and turned in for the shore with all possible speed. Long rose again, and fairly rolled down the hill in his eagerness to meet them. The launch touched the ice-foot, and the relief party hurried towards him. The ice-pilot of the *Bear* reached him first, spoke a word of cheer, and asked him where Greely was. He informed him of the location of the tent and the state of the party. They hurried in the direction indicated, and soon reached the tent, while Mr. Lowe took Long off to the *Bear*.

"In reply to our ice pilot's question, 'Is that you, Greely?' a feeble voice responded, 'Yes; cut the tent.' The pilot whipped out his knife and cut the hind end of the tent open from as high as he could reach to the ground Through this opening, Colwell entered. The light in the tent (it was 9 o'clock P. M.) was to dim to see plainly what lay before him, but he heard a voice in the farther corner, warning him to be careful and not step on Ellison and Connell. He found Greely lying under the folds of the tent, with the fallen poles across his body. Biederbeck was standing; Ellison and Connell lay on either side of the opening, the latter apparently dead. Stepping carefully across their bodies, he dragged Greely out and sat him up. He was so weak that he could barely swallow the crumbs of hard-tack that Colwell gave to him in the smallest pinches It was said that Greely first asked the rescuers if we were Englishmen; and on being told that we were his own countrymen, he added, 'and I am glad to see you.'"

